# Dr. Walker, in Answer to Mr. Ring

**Published:** 1806-06

**Authors:** J. Walker


### *To the Editors of the Medical and Physical Journal.*

Gentlemen,

*Salisbury Square*, 6 *v*, 1806.

I Feel myself thrown on the defensive by the criticisms of Mr. Ring, in your last number. He, there, speaks of an opinion 'highly dangerous' of 'dangerous doctrines,' &c. &c. There is a danger, of an extremely serious nature, attending the practice of vaccination, which I am afraid that he, himself, together with 'other labourers in the vineyard,' treats too lightly. It is the danger of exciting distresful alarm, and destroying that confiden c, which might be so easily afforded to the anxious parent, hanging upon the words of your mouth, as upon the sentence of an oracle, on the future lot of their darling offspring. Let not then the great champion of vaccination, fortifying, or rather encumbering himself "with all the instructions of all the vaccine societies, as well as of individuals on this point," endeavour to excite or increase our doubts, by declaring that this, or that that, is no test of security; let him tell us what is a certain test; let him give us a more simple one than the characteristic induration about about the tenth day, and I think he will render a considerable service to the cause; let him offer a more infallible one, and one that always presents itself, *Et is mihi erit magnus Apollo.*

A surgeon from Buckingham, happening to call, on my writing this, mentions a case to me where the inoculation was resisted till the twenty-ninth time, when a perfect Cow-pock was produced. If this had been a case of testing, the practitioner might have stopt at the twenty-seventh time, with some confidence, and afterwards have lost his patient by the small-pox. What assurance then can the resistance of a re-inoculation, *alone*, inspire? And when, in such case, are we to form our conclusion? As far, however, as the test of re-inoculation can be depended on, I must remain satisfied with the presence of the hardness at the part about the tenth day, as a true criterion; for I have never yet been able to produce the pock by re-inoculations after such appearance.

Perhaps the criterion I have offered might not have been so much censured, if I had called it a circumscribed characteristic hardness, ("a hardened phlegmonic base, and inflammatory areola encompassing the pock from the 9th to the 11th day,') and perhaps none would be more likely than Jenner himself, in his tremblingly alive precautions, (Vide vol. iii. page 502, lines 29 and 30,) to have pointed out other peculiarities; but these would not afford the desideratum which many medical men demand so earnestly. In presenting his discovery to the public, it was necessary to describe with minuteness and precision, the uninterrupted progress of the perfect pock; its cellular structure, firm and hard; its different successive shades of light pink, bluish, pearl, and dark mahogany colour; the areola an inch and a half or more in diameter, of a pink, scarlet, or crimson hue: it was necessary to point out the spurious pock; and all this has been done by the Medical Council of the Royal Jennerian Society. But the difference between the pock, evidently genuine, and the pustule altogether spurious, is so obvious that he who runs may read it: besides, while the people of every colour are interested in the Jennerian Inoculation, the above descriptions apply only to that smaller portion of them, the whites. What is most wanted now, is such description as shall enable us to seize on any invariable certain mark, when those which are liable from accident to be obliterated are absent. The induration at the inoculated part about the tenth day, is what appears to me to be a certain proof proof of the protecting effect being complete. This induration producing areola, while it is often so distinctly characterized from its circumscribed appearance, is, perhaps, not less so from its passing away quite differently from the induration and tumefaction, the consequences of injury, the irritation of the air, &c. &c. The successive changes do not take place at the outside, but coolness and softness soon succeed the inflammation, throughout the whole area, or field.

14, v. 1806. Since writing my Reply to Mr. Ring, I have perused the observations of Dr. Clarke, of Nottingham, in your last Journal, and find it requisite to make him the following explanation.—On receiving the letter he favoured me with, I informed him that an Editor of the Journal was present; that we were both exceedingly pressed for time at the moment, and that without waiting even to read it, I desired him to put it into his pocket, well supposing it to contain matter sufficiently important for the public eye. The learned Editors are wônt to apply their pruning knife to the productions of their immediate friends; with others they are not quite so unceremonious; and their admission since of the detail of the Proceedings of the Institution at Nottingham, may I hope have its use in many quarters of the empire.

On the opinion which I have offered, Dr. Clarke says, " it demands most serious consideration, that it is entirely new to him, presenting a perfect diagnosis between the true and what has been called the spurious cow-pox, &c." In further explanation let me add, that it sometimes happens when the sleeve is too straight, that the child, from its activity, or the adult, during his exertions in business, breaks the pock at an early period. The friction, which first broke it, continuing, we never see the perfectly formed pock; but, the characteristic inflammation, at the right time, shews that the complete effect has been produced. It sometimes happens that the evidently genuine pock being produced, from which there can be no objection to inoculate, the characteristic induration taking place at the right time, not a doubt remains but the protection has been complete, though the pock, in its last stages, exhibit very exactly the light coloured scab of a previous eruption (Vol. xii. page 45.) instead of the characteristically dark one. Thus then, I contend for it, that, whether your marks or tokens be swept off in the beginning, or in the passing away of that affection, which, I think, always merits, when at its height, the name of a disease, it it does at the time of its acme, and perhaps at no other period, give a certain and invariable proof of its presence, a certain and invariable sign of its efficacy.

Every one, much acquainted with the vaccine inoculation, must have noticed that there is often produced (from the wound of the lancet) in the centre of the pock, a considerable quantity of pus. Were I about to take matter from such pock, even as the beautiful one which is exhibited, through all its stages, in Ring's Treatise, I should think it right to remove the scab, and wipe out the little subjacent drop of pus, in order to guard against impurity; an earlier plate of Cuff, and one of Lee, in Pearson's Examination, &c. displaying also the small-pox, exhibit the cow-pock less depressed in its centre, and scarcely requiring such precaution. When the quantity of pus is very large, that of the vaccine matter in the vesicular or cellular ring which bounds it, is often in very small quantity; but, in the subject of whatever colour, or however affected, as I have frequently seen, with icthyosis, it will go its course, and duly exhibit the characteristic induration, though never the plump looking pock, nor subsequent *centrally* hard and full and firm scab. I have twice in Marmorice harbour, and once I think in London, seen all the appearance of incipient spurious pock, have the most satisfactory termination. Was it that a spurious kind of matter, a purulent matter as well as the vaccine, was on the point of my lancet, at the time of its application? or, was it that from the rude manner of my applying the instrument, early inflammation, discharge of serous fluid, and formation of pus were produced? Be this explicable how it may, some particle of the genuine virus had been laid hold of, went on silently and secretly to produce its effect, and though amid the local derangement just described, it could not exhibit the uninterrupted and beautifully regular pock, it did in right time produce the erythematous inflammation, with the circumscribed characteristic hardness, leaving no doubt of the protection being complete.

There is a statement in Dr. Clarke's letter, that I fear may convey an idea to your readers, which he cannot have intended, viz. that from the induration, with the other usual marks, I should have pronounced the protection complete. At the time of the induration, I never saw the child, and on the certainty which the cicatrix affords, my notion is but too strongly expressed in page 55, vol. xiii. where, in line 14, for are left, I ought to have said, are sometimes sometimes left. It is worthy of notice, however, that from the case which I have no distinct recollection of, but on which Mr. Drew says (547, vol. xiv.) I had, like himself, been sanguine, &c. this latter gentleman inoculated three patients with great attention, without being able to produce small-pox.

If you can afford room for it, I take the present opportunity to offer a short reply to another of your respectable correspondents, who in vol. xiii, 542, says, they understood at Plymouth, that several cases of secondary variolæ had occurred in subjects who had been vaccinated at Head Quarters. If by Head Quarters, he mean the Metropolis of the Empire, certain late publications may shew that all kinds of mistakes are committed, all kinds of misrepresentations are fabricated, in it. If he means the Royal Jennerian Society, he ought to know, that only one of its stations is open every day; that if, at the others, any patient have not regularly attended till assured of the protection being complete, the Vaccinator is not to blame; that to the vaccinated, who by thousands have had their pocks on each arm broken down from day to day, and as far as possible completely exhausted at the Central House, (Vol. XIII. p. 456, l. 3, 4, *a fundo)* for the supply of happily unceasing applications from all quarters, the shield of Jenner has been found their sure defence against the poisoned arrows of contagion, which have of late been flying so thick through every lane and alley of London. The remarks of Mr. Dunning, on the *cicatrices, sequelæ,* (we do not in our language, like the Germans, Dutch, &c. give the different governed cases of Latin words) of inoculation, are, I think, extremely correct, and likely to be useful to many. They perfectly correspond with the observations of some accurate inoculators in town; and their publication, I think, may help pretty strongly and fairly to confirm the conclusion, in many cases, that the vaccination has been complete. On the other hand, what he says respecting 'the ulcerative or purulent process, succeeding early rupture of the pock, and leaving even deep and extensive cicatrices, may, I think, tend to induce a most wholesome caution in those who do not attend to the presence of the characteristic induration as the criterion. If ulceration take place to the prevention of the areola, we have no right to conclude that vaccination has been effected. The work was begun, and a local effect was produced by it, but the ulcerated process prevented its entering or acting upon the system, prevented its producing any protection whatsoever. J. WALKER.

